# The Morphological Spectrum of Endometrial Biopsies in Nigerians: A Snapshot of a Review of Findings From a District Hospital

**DOI:** 10.7759/cureus.79239

**Published:** 2025-02-18

**Authors:** Ijeoma A Okwudire-Ejeh, Kevin N Ezike, Aminu M Mai, Shiktira D Kwari, Oluseyi A Asaolu, Umar M Umar, Bamnan C Dallang, Emmanuel E Oguntebi

**Affiliations:** 1 Anatomic Pathology and Forensic Medicine, Asokoro District Hospital, Abuja, NGA; 2 Anatomic Pathology and Forensic Medicine, Nile University of Nigeria, Abuja, NGA; 3 Obstetrics and Gynaecology, Nile University of Nigeria, Abuja, NGA; 4 Obstetrics and Gynaecology, Wuse District Hospital, Abuja, NGA; 5 Radiology, Nile University of Nigeria, Abuja, NGA

**Keywords:** abnormal uterine bleeding, endometrial biopsies, endometrial polyps, nigeria, products of conception

## Abstract

Introduction

Endometrial biopsies constitute a significant proportion of the work of pathologists. They are associated with a wide spectrum of diagnoses, including inflammatory, non-neoplastic, and neoplastic lesions. They are an invaluable procedure used by gynecologists in conjunction with pathologists to assess endometrial health. This study aimed to document the morphological spectrum of endometrial biopsies in Nigerians, compare these with findings in other populations, and establish baseline data that may aid in health systems planning and directing resource allocation for improvement in women's health.

Materials and methods

This was an eight-year retrospective study of all endometrial biopsy samples received in the anatomic pathology and forensic medicine department of Asokoro District Hospital, Abuja, Nigeria. Hospital records and surgical pathology reports were retrieved for patients’ biodata and clinical information. Appropriate slides were retrieved and reviewed, and fresh sections were made where necessary. We categorized the lesions as non-neoplastic, gestational trophoblastic disease (GTD), non-GTD neoplastic, normal, and others. The proliferative and neoplastic lesions were classified using the 2020 WHO classification of female genital tumors. Malignant lesions were also graded using the 2009 FIGO guidelines. Data obtained were analyzed, and results were presented as percentages/frequencies and displayed as tables and charts.

Results

A total of 1,506 cases met the inclusion criteria (age range: 13-84 years, median age: 34 years). The majority of cases (87.6%, 1319/1506) occurred in patients in the reproductive age range of 15-44 years, with 62.7% (945/1506) in the range of 34-44 years. Cases in patients aged 45 years and above constituted just 12.4% (186/1506). The most frequently reported symptoms were abnormal uterine bleeding (AUB) (42.2%, 635/1506), abdominal pain (10.7%, 161/1506), and amenorrhea (8.8%, 133/1506). Non-neoplastic lesions (79.4%, 1196/1506) dominated the diagnostic spectrum, and of these, 80.4% (961/1196) were placental (products of conception). The frequency of non-placental, non-neoplastic lesions was 19.6% (235/1196). They were mainly endometrial polyps (52.8%, 124/235), seen mostly (50.8%, 63/124) in the age range of 35-44 years. Inflammatory lesions (10.6%, 25/235) were least frequently diagnosed, occurring mostly (48.0%, 12/25) in the age range of 15-29 years. GTDs comprised 5.0% (75/1506) of total lesions, with 92.0% (69/75) of these patients younger than 45 years. Hydatidiform moles (88.0%,66/75) constituted the majority, while choriocarcinoma, the only malignant GTD diagnosed, occurred in 10.6% (8/75). Neoplastic lesions constituted 4.2% (63/1506) of cases and 76.2% (48/63) of mesenchymal leiomyomas. All the epithelial neoplasms (13/63, 20.6%) were malignant, occurring in 92.3% (12/13) of patients above 44 years.

Conclusions

Our study demonstrated significant similarities in the morphological spectrum of endometrial biopsies between Nigerians and people of other nationalities. These lesions were predominantly non-neoplastic and seen mostly in the reproductive age range, while lesions in peri and postmenopausal patients were fewer and more likely to be malignant. The predominance of products of conception signifying an unacceptably high rate of early pregnancy loss and a high percentage of endometrial polyps within the non-pregnancy-related biopsies warrant further research.

## Introduction

Endometrial biopsy refers to the procedure of sampling the innermost lining of the uterine corpus, the endometrial mucosa, through a surgical procedure for diagnostic and therapeutic purposes [1,2]. Endometrial biopsy specimens are among the most commonly encountered surgical specimens. However, they pose a diagnostic challenge to the pathologist as they are associated with a wide variety of alterations [2,3]. There are different methods of obtaining these biopsies, including dilatation and curettage (D & C), aspiration (office biopsy), and hysteroscopy methods [3]; and the choice of the method depends on the indication and/or the clinical circumstances.

Indications for endometrial biopsy or curettage are classified into major and minor; major indications include the determination of the cause of abnormal uterine bleeding (AUB), evaluation of the status of the endometrium in infertile patients, including histologic dating, evacuation of products of conception, either spontaneous abortions or termination of pregnancy, assessment of the response of the endometrium to hormonal therapy, especially estrogen replacement in perimenopausal and postmenopausal women and Tamoxifen therapy for breast cancer [2,4]. The indications considered minor include atypical or abnormal glandular cells of undetermined significance (AGUS) diagnosed in a cervical-vaginal cytologic specimen that requires endometrial sampling to exclude hyperplasia or carcinoma and when transvaginal ultrasound shows a thickened endometrial lining in postmenopausal patients [2,4].

The procedure is relatively safe and has a high diagnostic yield; however, complications, when they occur, include, most commonly, cramping and light vaginal bleeding or spotting for several days and, less commonly, uterine perforation, pelvic infection, and bacteremia [4]. Contraindications to performing an endometrial biopsy are either absolute, including pregnancy, acute pelvic inflammatory disease, acute cervicovaginal infection, cervical cancer, and patient refusal; or relative, including morbid obesity, cervical stenosis, and clotting disorders or coagulopathies [4].

This study aims to document and analyze the diagnoses of endometrial biopsies at Asokoro District Hospital, Abuja, Federal Capital Territory (FCT), Nigeria, to establish baseline data that will hopefully foster and enable overall improvements in health systems management and specifically direct resource allocation for improvement in women's health. Several studies on this topic have been conducted in other parts of Nigeria and the world, and the findings of this study will also be compared with those. To the best of our knowledge, only one similar study has been published from Abuja, the location of this particular work, and it was limited only to endometrial biopsies for the evaluation of AUB [5]. Our center caters to a wider area than the one in the earlier study [5]. Hence, our study applies broader inclusion criteria to achieve its goal, which is to provide a potentially generalizable "snapshot" of the morphological spectrum of endometrial biopsies, and their relative frequency in Nigerians. The higher number of endometrial biopsies performed in the study also enabled the inclusion of a larger sample.

## Materials and methods

Study setting

This was a retrospective study of all endometrial biopsies received in the anatomic pathology and forensic medicine department of Asokoro District Hospital, Abuja, Federal Capital Territory (FCT), Nigeria, over eight years: from January 1, 2015 to December 31, 2022. Asokoro District Hospital is a quasi-tertiary center situated in Abuja, FCT, North Central Nigeria. It is a cosmopolitan center and is uniquely positioned as it houses the only fully equipped histopathology laboratory that serves 13 other sister hospitals run by the Federal Capital Territory Administration (FCTA); and accepts samples from many other hospitals from within the FCT and other neighboring states like Kogi, Nasarawa, and Niger States. Furthermore, Abuja, being Nigeria’s capital city and the seat of its government, is a melting pot and home to people from various tribes and ethnic nationalities that make up Nigeria [6]. Thus, the sample size and selection are expected to be reasonably representative of the spectrum of different diagnoses and acceptably representative of their relative frequency in the Nigerian population.

Study design

The surgical pathology reports of all endometrial biopsy samples received during the study period were retrieved from the departmental records, while data on the patient’s biodata and clinical features were obtained from the hospital’s electronic archives. The appropriate slides were retrieved and reviewed. In cases where the review of retrieved slides or the new sections cut to replace missing slides resulted in a different diagnosis, the protocol for informing the managing clinician and patient would be implemented. All biopsies had been fixed in 10% neutral buffered formalin.

Inclusion and exclusion criteria

Inclusion Criteria

Endometrial biopsy cases, obtained via curettage, hysteroscopy, or aspiration, with complete biodata and clinical information, and for which adequately informative glass slides were available, were selected for the study. Cases where the glass slides were not found but their FFPE tissue blocks were available, and new H&E slides were produced, were also selected.

Exclusion Criteria

Cases lacking complete biodata, clinical information, or both glass slides and FFPE tissue blocks were excluded from the study. Additionally, cases where the endometrium was evaluated as part of hysterectomy samples were also excluded.

Diagnostic criteria

The diagnostic spectrum was categorized as follows: normal; non-neoplastic (placental conditions, inflammatory and proliferative); gestational trophoblastic diseases (GTDs); non-GTD neoplastic (epithelial, mixed, and mesenchymal); and others. The proliferative and neoplastic lesions were classified according to the 2020 WHO classification of female genital tumors [7]. The malignant neoplastic lesions were also graded using the 2009 FIGO guidelines [8].

Statistical analysis

All data were entered using the spreadsheet of Microsoft Office 365 version of the Excel software program (Microsoft Corporation, Redmond, WA) and analyzed with SPSS Statistics for Windows, version 23.0 (IBM Corp., Armonk, NY). Continuous variables were summarized using range and mean ± standard deviation (SD), while categorical variables presented as percentages/frequencies were determined using descriptive statistics. Results were displayed using tables and charts.

Ethical consideration

Ethical clearance was obtained from the Medical Ethics Committee of Asokoro District Hospital (approval no: FCTA/HHSS/HMB/ADH/138/23).

## Results

**Figure 1:**
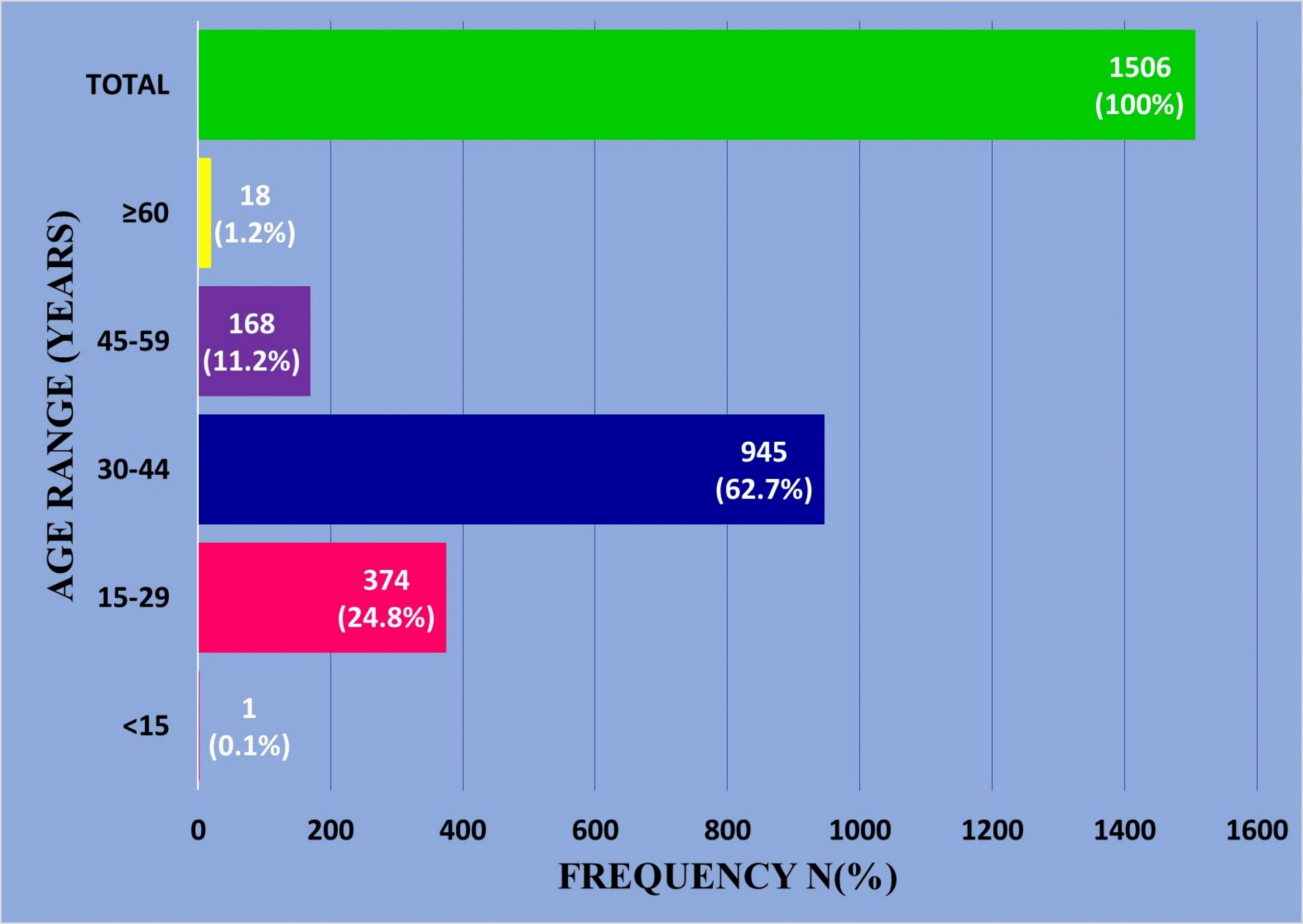
Age distribution of patients with endometrial lesions

A total of 1506 endometrial biopsy cases received during the study period met the inclusion criteria. The mean age of the patients was 35.1 years (range: 13-84 years), while the median age was 34 years. The peak range was 30-44 years (62.7%, 945/1506). The least number of patients were in the pre and perimenarche (0.1%, 1/1506) and peri and post-menopause (1.2%, 18/1506) age ranges (Figure 1).

The most common symptoms reported were AUB (42.2%, 635/1506), abdominal pain (10.7%, 161/1506), amenorrhea (8.8%, 133/1506), infertility (5.2%, 78/1506), and irregular menstruation (2.8%, 42/1506). In terms of diagnostic spectrum, most cases (79.4%, 1196/1506) were non-neoplastic lesions; the majority of these (80.4%, 961/1196) were products of conception. Non-neoplastic lesions overwhelmingly occurred in patients aged over 15 years and younger than 45 years (92.1%, 1102/1196).

Neoplastic lesions, comprising GTDs and non-GTD lesions, were the least prevalent, accounting for 9.2% (138/1506) of cases. The GTDs occurred more frequently in patients of reproductive age, while the non-GTD epithelial neoplastic lesions were seen more frequently in perimenopausal and menopausal patients. The only biopsy specimen received from a patient aged under 15 years was diagnosed as normal proliferative phase endometrium (Table 1).

**Table 1:** Diagnosis and age distribution of patients with endometrial lesions AH/EIN: atypical hyperplasia/endometrial intraepithelial neoplasia; FIGO: International Federation of Gynecology and Obstetrics; GTD: gestational trophoblastic disease

Category	Diagnosis	Age range, years					Total, n (%)
		<15, n (%)	15-29, n (%)	30-44, n (%)	45-59, n (%)	≥60, n (%)	
Non-neoplastic	Inflammatory						1196 (79.4)
	Acute endometritis	0 (0.0)	10 (0.7)	8 (0.5)	0 (0.0)	0 (0.0)	
	Chronic endometritis	0 (0.0)	2 (0.1)	3 (0.2)	1 (0.1)	0 (0.0)	
	Granulomatous endometritis	0 (0.0)	0 (0.0)	1 (0.1)	0 (0.0)	0 (0.0)	
	Proliferative						
	Endometrial polyps	0 (0.0)	16 (1.1)	63 (4.2)	43 (2.9)	2 (0.1)	
	Hyperplasia without atypia	0 (0.0)	11 (0.7)	29 (1.9)	17 (1.1)	2 (0.1)	
	AH/EIN	0 (0.0)	1 (0.1)	6 (0.4)	7 (0.5)	0 (0.0)	
	Endocervical polyp	0 (0.0)	1 (0.1)	5 (0.3)	7 (0.5)	0 (0.0)	
	Placental conditions						
	Products of conception	0 (0.0)	280 (18.6)	666 (44.2)	15 (1.0)	0 (0.0)	
GTD	Partial hydatidiform mole	0 (0.0)	14 (0.9)	15 (1.0)	3 (0.2)	0 (0.0)	75 (5.0)
	Complete hydatidiform mole	0 (0.0)	12 (0.8)	19 (1.3)	3 (0.2)	0 (0.0)	
	Choriocarcinoma	0 (0.0)	2 (0.1)	6 (0.4)	0 (0.0)	0 (0.0)	
	Exaggerated implantation site	0 (0.0)	0 (0.0)	1 (0.1)	0 (0.0)	0 (0.0)	
Non-GTD neoplastic	Leiomyoma	0 (0.0)	3 (0.2)	38 (2.5)	7 (0.5)	0 (0.0)	63 (4.2)
	Endometrioid carcinoma FIGO grade 2	0 (0.0)	0 (0.0)	1 (0.1)	2 (0.1)	4 (0.3)	
	Endometrioid carcinoma FIGO grade 3	0 (0.0)	0 (0.0)	0 (0.0)	3 (0.2)	0 (0.0)	
	Nonkeratinizing squamous cell carcinoma	0 (0.0)	0 (0.0)	0 (0.0)	2 (0.1)	0 (0.0)	
	Malignant mixed Mullerian tumor	0 (0.0)	0 (0.0)	0 (0.0)	0 (0.0)	1 (0.1)	
	Undifferentiated uterine sarcoma	0 (0.0)	0 (0.0)	0 (0.0)	1 (0.1)	0 (0.0)	
	Serous carcinoma	0 (0.0)	0 (0.0)	0 (0.0)	0 (0.0)	1 (0.1)	
Normal	Proliferative	1 (0.1)	5 (0.3)	13 (0.9)	10 (0.7)	0 (0.0)	87 (5.8)
	Secretory	0 (0.0)	7 (0.5)	21 (1.4)	3 (0.2)	0 (0.0)	
	Menstrual	0 (0.0)	0 (0.0)	4 (0.3)	0 (0.0)	0 (0.0)	
	Atrophy	0 (0.0)	0 (0.0)	0 (0.0)	19 (1.3)	4 (0.3)	
Others	Descriptive	0 (0.0)	4 (0.3)	15 (1.0)	17 (1.1)	2 (0.1)	85 (5.6)
	Exogenous hormone use	0 (0.0)	4 (0.3)	9 (0.6)	1 (0.1)	0 (0.0)	
	Blood clot	0 (0.0)	0 (0.0)	6 (0.4)	4 (0.3)	1 (0.1)	
	Uterine synechiae	0 (0.0)	2 (0.1)	9 (0.6)	0 (0.0)	0 (0.0)	
	Osseous metaplasia	0 (0.0)	0 (0.0)	8 (0.5)	0 (0.0)	0 (0.0)	
	Inconclusive	0 (0.0)	0 (0.0)	0 (0.0)	2 (0.1)	1 (0.1)	
Total		1 (0.1)	374 (24.8)	945 (62.7)	168 (11.2)	18 (1.2)	1506 (100)

**Figure 2:**
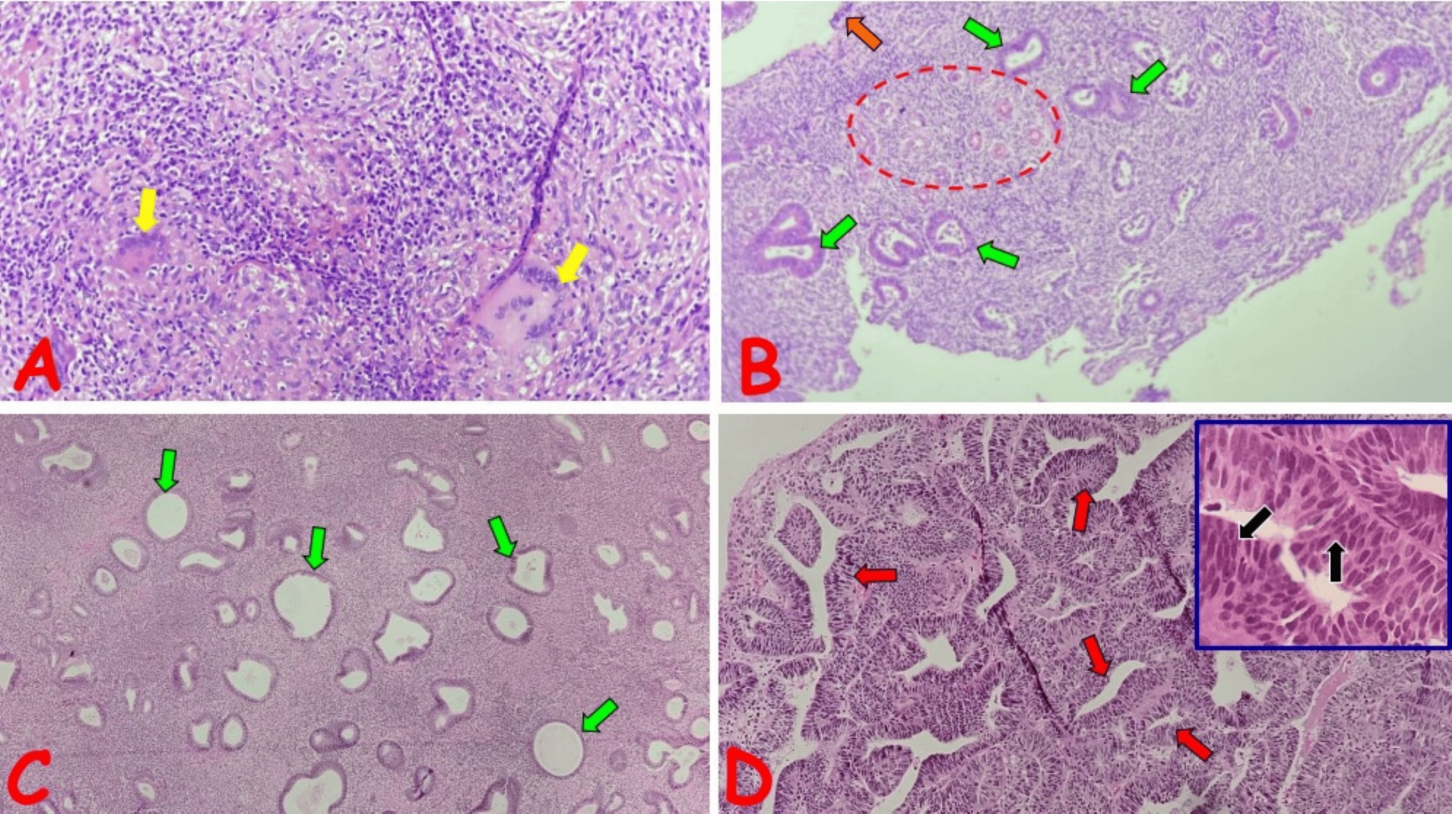
Photomicrographs of selected non-neoplastic endometrial lesions (A) Chronic granulomatous inflammation of the endometrium in a 39-year-old female with a history of primary infertility; note the non-caseating granulomas, composed of sheets of epithelioid macrophages surrounded by lymphocytes, and containing Langhans-type inflammatory multinucleated giant cells (yellow arrows). (B) Endometrial polyp in a 35-year-old female with a history of AUB; note the benign polypoid lesion partially covered by attenuated columnar epithelium (orange arrow) which overlies compact stroma containing clusters of thick-walled vascular channels (red circle) and frequently dilated, benign, endometrial glands (green arrows). (C) Endometrial hyperplasia without atypia in a 39-year-old female with a history of AUB; note the increased gland-to-stroma ratio and frequent foci of cystically dilated glands (green arrows). (D) Endometrial hyperplasia with atypia in a 42-year-old female with a history of AUB; note the markedly increased gland-to-stroma ratio with closely packed glands leaving little intervening stroma (red arrows). Inset shows atypical gland exhibiting stratification and loss of polarity of hyperchromatic nuclei (black arrows) AUB: abnormal uterine bleeding

The age distribution among patients with non-pregnancy-related non-neoplastic conditions was statistically significant. Non-pregnancy-related non-neoplastic conditions made up 19.6% (235/1196) of cases. Of these, endometrial polyps (52.8%, 124/235) were the most frequently diagnosed. They were most prevalent in patients in the age range of 30-44 years, who accounted for 50.8% (63/124) of cases. Inflammatory lesions (10.6%, 25/235) were the least frequently diagnosed and were most prevalent in the age range of 15-29 years (48.0%, 12/25) (Table 2, Figure 2).

**Table 2:** Age distribution of patients with non-neoplastic endometrial lesions Chi-square: 32.445; p=0.019 (significant at 95%) AH/EIN: atypical hyperplasia/endometrial intraepithelial neoplasia

Diagnosis	Age range, years					Total. n (%)
	<15, n (%)	15-29, n (%)	30-44, n (%)	45-59, n (%)	≥60, n (%)	
Inflammatory						
Acute endometritis	0 (0.0)	10 (0.8)	8 (0.7)	0 (0.0)	0 (0.0)	18 (1.5)
Chronic endometritis	0 (0.0)	2 (0.2)	3 (0.3)	1 (0.1)	0 (0.0)	6 (0.5)
Granulomatous	0 (0.0)	0 (0.0)	1 (0.1)	0 (0.0)	0 (0.0)	1 (0.1)
Proliferative						
Endometrial polyps	0 (0.0)	16 (1.3)	63 (5.3)	43 (3.6)	2 (0.2)	124 (10.4)
Hyperplasia without atypia	0 (0.0)	11 (0.9)	29 (2.4)	17 (1.4)	2 (0.2)	59 (4.9)
AH/EIN	0 (0.0)	1 (0.1)	6 (0.5)	7 (0.6)	0 (0.0)	14 (1.2)
Endocervical polyp	0 (0.0)	1 (0.1)	5 (0.4)	7 (0.6)	0 (0.0)	13 (1.1)
Placental conditions						
Products of conception	0 (0.0)	280 (23.4)	666 (55.6)	15 (1.3)	0 (0.0)	961 (80.4)
Total	0 (0.0)	321 (26.8)	781 (65.3)	90 (7.5)	4 (0.3)	1196 (100.0)

**Figure 3:**
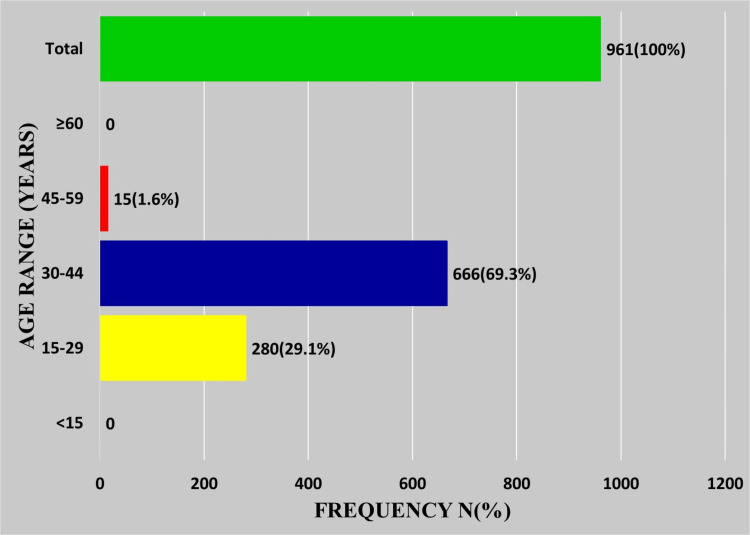
Age distribution of patients with placental conditions (products of conception)

**Figure 4:**
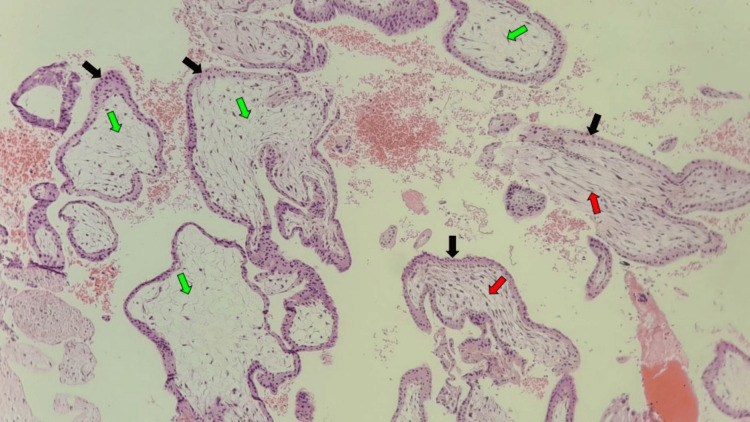
Photomicrograph of products of conception Products of conception in a 27-year-old female with a history of vaginal bleeding and lower abdominal pain following eight weeks of amenorrhea; note the variably sized chorionic villi with admixture of lax (green arrows) and compact fibrous stroma (red arrows), lined by trophoblasts

Placental conditions, diagnosed as products of conception, were by far the most common non-neoplastic lesions, accounting for 80.4% (961/1196). Not surprisingly, the majority of the patients (98.4%, 946/961) were in the reproductive age range of 15-44 years (Figures 3, 4).

**Figure 5:**
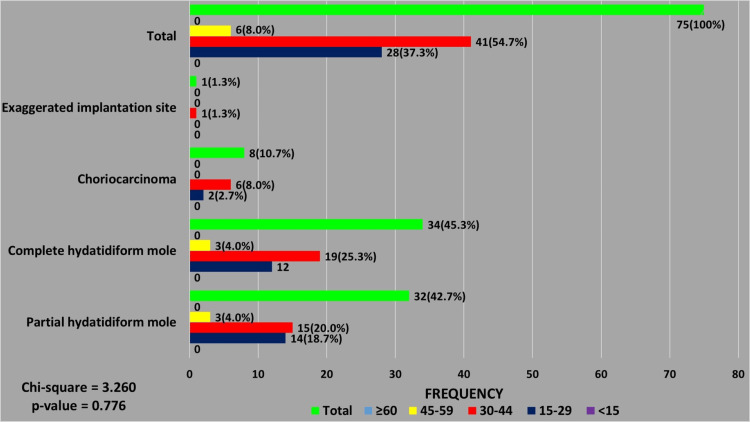
Age distribution of patients with gestational trophoblastic diseases

**Figure 6:**
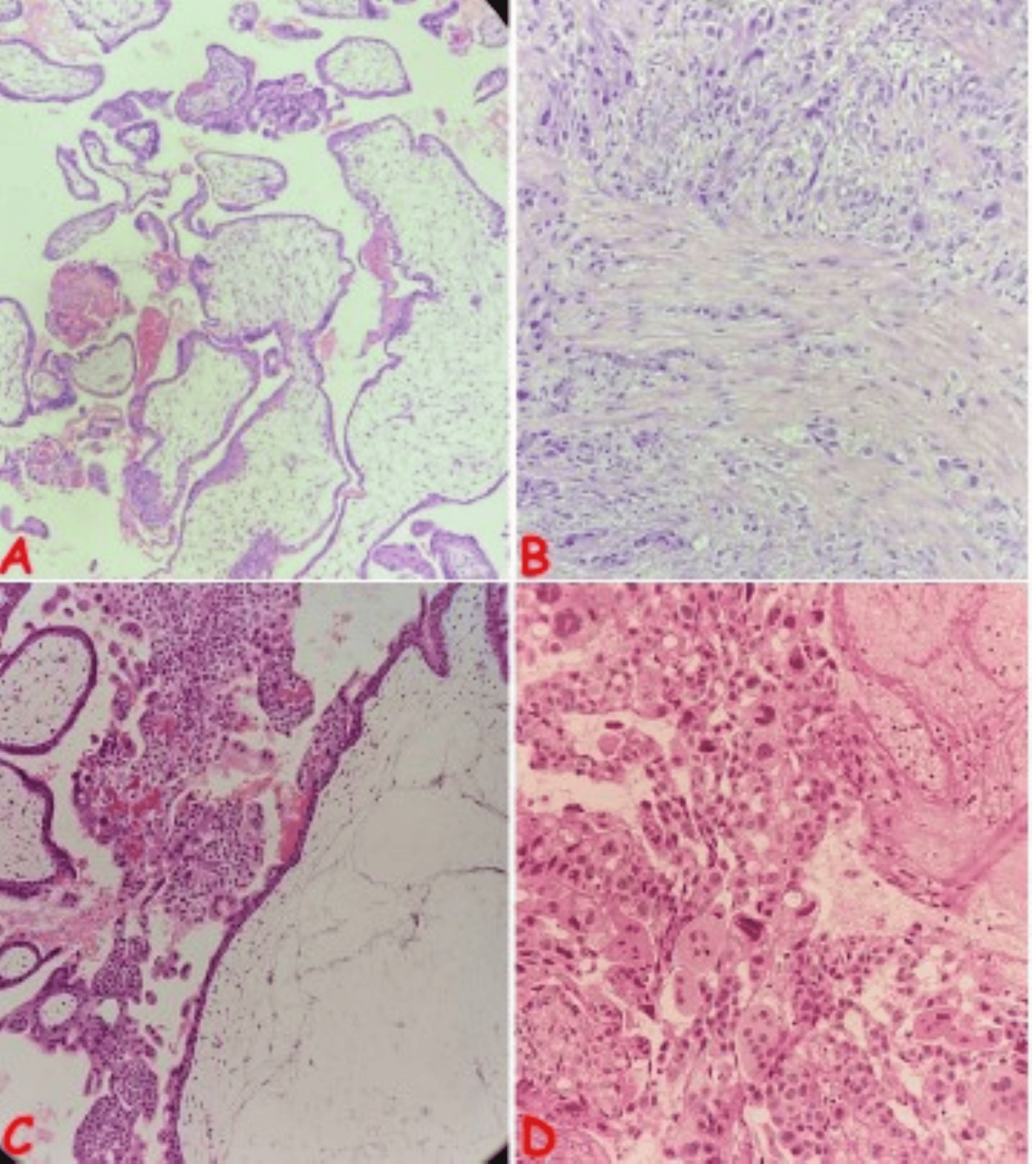
Photomicrographs of selected gestational trophoblastic disease cases (A) Partial hydatidiform mole in a 36-year-old multiparous female with a history of recurrent spontaneous abortions; note the mixed population of hydropic (yellow arrows) and normal (red arrows) chorionic villi. (B) Exaggerated placental site in a 21-year-old female with a history of vaginal bleeding following incomplete abortion; note the foci of implantation site-type intermediate trophoblasts (green arrows) invading the myometrium (black arrows). (C) Complete hydatidiform mole in a 28-year-old female with a history of vaginal bleeding following three months of amenorrhea; note the massively dilated chorionic villus with cistern formation (red arrow), marked trophoblastic hyperplasia with atypia (blue arrow), and hydropic chorionic villi (yellow arrow). (D) Choriocarcinoma in a 36-year-old female with a previous history of molar pregnancy; note the triphasic proliferation consisting of atypical syncytiotrophoblasts, cytotrophoblasts, and intermediate trophoblasts, exhibiting bizarre cytology, including multinucleation (blue arrows) with areas of necrosis (black arrow)

GTDs comprised 5.0% (75/1506) of the total lesions, with 92.0% (69/75) seen in patients younger than 45 years. Molar pregnancies, including complete and partial hydatidiform moles (88.0%, 66/75) formed the majority of these GTDs. Complete hydatidiform moles, (51.5%, 34/66) were slightly more frequently diagnosed than partial moles (48.5%, 32/66). Combined, the majority of cases of hydatidiform mole (90.9%, 60/66) occurred in patients in the reproductive age range of 15-44 years. A single case of an exaggerated placental site was diagnosed in a patient aged 21 years. Choriocarcinoma was the only malignant GTD diagnosed and accounted for 10.6% (8/75) of all GTDs. The majority of these patients (62.5%, 5/8) were multiparous; 37.5% (3/8) had a history of previous spontaneous abortions, and one of them had a prior diagnosis of molar pregnancy. In terms of age distribution, 75.0% (6/8) of these choriocarcinoma cases were seen in patients in the age range of 30-44 years. Four of these patients were aged between 31 and 36 years, while the two patients above 40 years were aged 41 and 44 years, respectively (Figures 5, 6).

**Figure 7:**
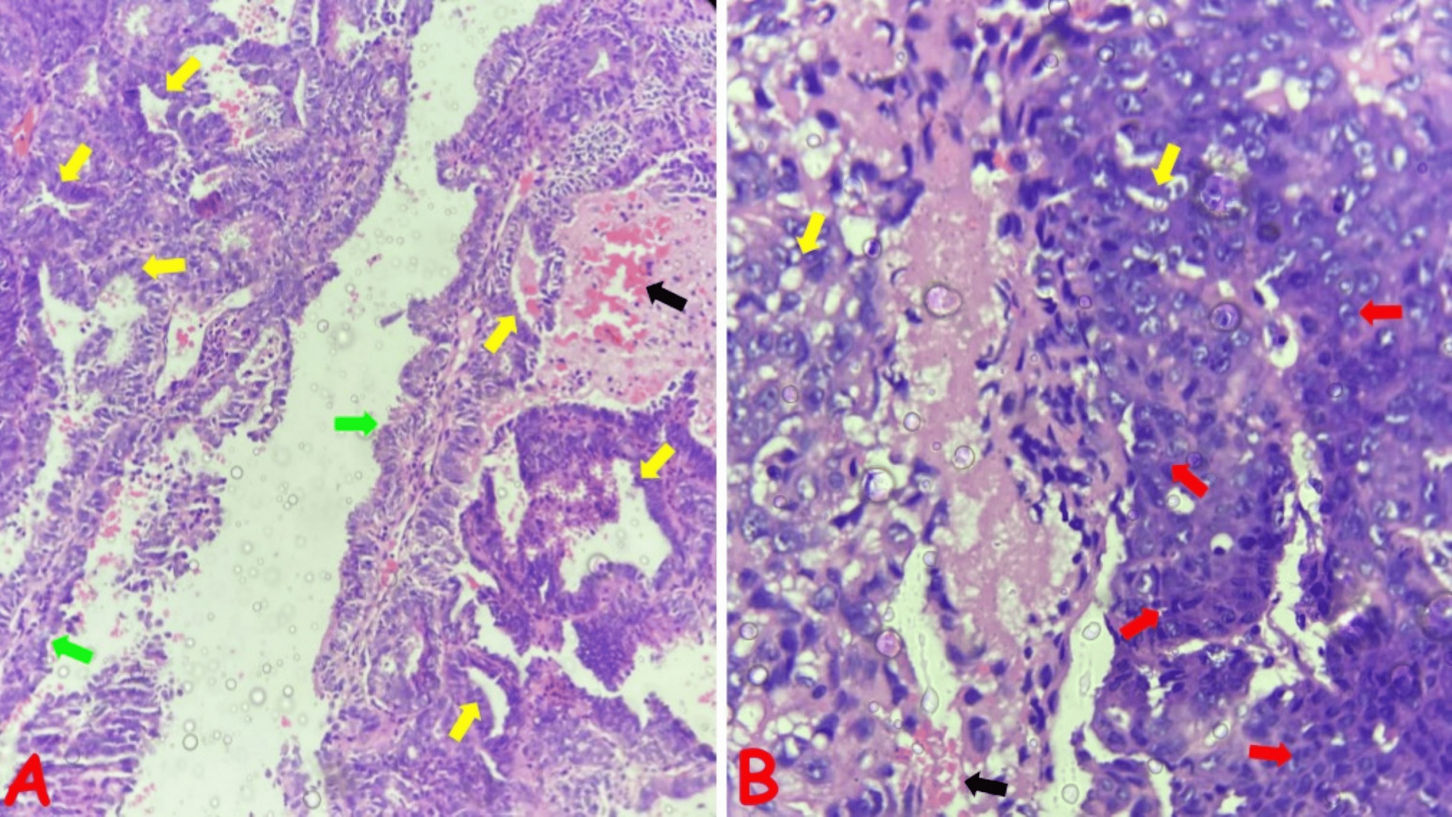
Photomicrographs of selected non-GTD neoplastic endometrial lesions (A) Endometrioid endometrial carcinoma, FIGO grade 2, in a 62-year-old, postmenopausal female with a history of postmenopausal bleeding; note the variably sized atypical glands (yellow arrows) and papillary structures with delicate fibrovascular core (green arrows). (B) Endometrioid endometrial carcinoma, FIGO grade 3, in a 57-year-old, postmenopausal female with a five-year history of AUB; note the solid sheets of atypical cells with vesicular nuclei constituting more than 50% of tumor (red arrows) and occasional foci of atypical glands (yellow arrows) AUB: abnormal uterine bleeding; FIGO: International Federation of Gynecology and Obstetrics; GTD: gestational trophoblastic disease

The overall frequency of neoplastic lesions was 4.2% (63/1506), of which leiomyomas (76.2%, 8/63) were the most frequent. All the epithelial neoplastic lesions (13/63, 20.6%) were malignant, occurring mostly (92.3%, 12/13) in patients aged above 44 years. In contrast, most of the mesenchymal neoplastic lesions (98.0%, 48/49) were benign leiomyomas and occurred predominantly (85.4%, 41/48) in patients aged below 45 years (Table 3, Figure 7).

**Table 3:** Age distribution of patients with non-GTD neoplastic endometrial lesions Chi-square: 63.414; p<0.001 (significant at 95%) FIGO: International Federation of Gynecology and Obstetrics; GTD: gestational trophoblastic disease

Diagnosis	Age range, years					Total, n (%)
	<15 n (%)	15–29 n(%)	30–44 n(%)	45–59 n(%)	≥60 n(%)	
Leiomyoma	0 (0.0)	3 (6.3)	38 (79.2)	7 (14.5)	0 (0.0)	48 (76.2)
Endometrioid carcinoma FIGO grade 2	0 (0.0)	0 (0.0)	1 (14.3)	2 (28.6)	4 (57.1)	7 (11.1)
Endometrioid carcinoma FIGO grade 3	0 (0.0)	0 (0.0)	0 (0.0)	3 (100.0)	0 (0.0)	3 (4.7)
Nonkeratinizing squamous cell carcinoma	0 (0.0)	0 (0.0)	0 (0.0)	2 (100.0)	0 (0.0)	2 (3.2)
Malignant mixed Mullerian tumor	0 (0.0)	0 (0.0)	0 (0.0)	0 (0.0)	1 (100.0)	1 (1.6)
Serous carcinoma	0 (0.0)	0 (0.0)	0 (0.0)	0 (0.0)	1 (100.0)	1 (1.6)
Undifferentiated uterine sarcoma	0 (0.0)	0 (0.0)	0 (0.0)	1 (100.0)	0 (0.0)	1 (1.6)
Total	0 (0.0)	3 (6.3)	39 (61.9)			63 (100)

## Discussion

The most frequent indication for endometrial biopsy in our study was AUB from retained products of conception. This aligns with the findings reported by Asuzu et al.; Kurt et al., in Turkey, who reviewed only non-obstetric endometrial biopsies, also reported AUB as the most frequent indication. However, our results differ from the findings of Abdullahi et al. in South West Nigeria, who reported infertility as the most frequent indication [5,9,10]. The age range of patients in this study clustered in the reproductive age range of 15-44 years, similar to the studies by Asuzu et al. and Vhriterhire et al.; hence, the majority of the diagnoses were pregnancy-related, including products of conception and GTD [5,11].

The diagnostic spectrum of endometrial lesions in our study differed in frequency by age range rather than symptomatology. The non-neoplastic lesions were more frequent in the reproductive age group, while the neoplastic lesions occurred more frequently in the perimenopausal and menopausal age groups. These findings are similar to those from other Nigerian studies - Asuzu et al., Vhriterhire et al., and Dike et al. - and studies from other regions of the world: Husain et al., Kurt et al., and Siddiqui et al. [5,9,10,11-13]. The reasons for the higher frequency of products of conception in the age range of 35-44 than in that of 15-34 years are outside the scope of this study; however, the etiology of pregnancy loss, especially in the early weeks, is mainly due to chromosomal abnormalities, and increasing maternal age is a major risk factor for the development of these chromosomal abnormalities [14,15].

Non-pregnancy-related non-neoplastic conditions (19.6%) were a common finding, second only to pregnancy-related conditions, and of these, endometrial polyps accounted for a majority of the diagnoses. These findings are similar to those reported by Asuzu et al. in North Central Nigeria, Husain et al. in Saudi Arabia, and Mowers et al. in the USA [5,16,17]. The precise etiology of endometrial polyps is unknown; however, it is associated with endometrial hyperplasia and increased and/or unopposed estrogen secretion [18]. Many studies in Europe, India, and the USA report a higher frequency of endometrial polyps in peri and postmenopausal patients [19-21]. On the other hand, our study and other Nigerian and Saudi Arabian studies report a higher frequency in premenopausal patients [5,16,22]. The reasons for these differences are unclear, but sampling bias and study design are possible factors. Nevertheless, more studies are required to rule out genetic and/or racial factors.

Non-pregnancy-related inflammatory lesions - acute, chronic, and granulomatous endometritis - were not as frequent (10.6%) in our study as other non-pregnancy-related non-neoplastic lesions, similar to the findings by Dike et al. (12.2%), but higher than those reported by Asuzu et al. (4.0%), Abdullahi et al. (7.8%), Husain et al. (4.8%), Siddiqui et al. (3.1%), Lahari et al. (3.4%), and Forae et al. (1.3%); and much lower than those documented by Song et al. (24%), most probably because Song et al. used very broad criteria of one plasma cell identified per 10 high-power fields, and immunohistochemistry for the diagnosis of endometritis [5,10,16,21-23]. The majority of these inflammatory lesions occurred in patients in the reproductive age range (15-44), similar to the findings of Asuzu et al., Dike et al., Siddiqui et al., Husain et al., and Abubakar et al. [5,9,10,16,24].

GTDs are a diverse group of diseases associated with normal or abnormal gestation and characterized by proliferation of trophoblasts and elevation of the β subunit of human chorionic gonadotropin (βHCG) [25,26]. The global incidence of GTD exhibits notable variations [27,28]. Most of the data coming out from Nigeria, which indicate a higher incidence than other parts of the world, are from hospital-based studies; and incidence rates, extrapolated as they are from these hospital-based studies, may be skewed by study limitations, including methodology, insufficient records of pregnancies (viable and aborted), and high variability of pregnancy rates between Nigeria and other regions of the world [26]. Nevertheless, the GTD frequency of 5% in our study is similar to the findings of other Nigerian studies, including those by Asuzu et al. (4.7%), Abdullahi et al. (5.0%), and Abubakar et al. (5.0%) [5,10,24]. Dike et al. (2.7%) and Forae et al. (1.7%), who reported lower frequencies, had much smaller sample sizes than the aforementioned studies [12,22].

The risk factors for GTD include parity, higher maternal age, history of previous and/or recurrent spontaneous abortions, and history of previous molar pregnancy, which were evident in the majority of our GTD patients [25-27]. The disease spectrum of GTDs includes hydatidiform mole (complete and incomplete), invasive mole, choriocarcinoma, placental site trophoblastic tumor, and miscellaneous trophoblastic lesions [27]. In keeping with the findings of other studies from Nigeria and other regions of the world, our study revealed hydatidiform moles to be the most frequently diagnosed GTD [5,10,12,22,24,26,27].

The spectrum of non-GTD neoplastic lesions of the endometrium includes epithelial lesions such as endometrial intraepithelial neoplasia (EIH), endometrial carcinoma variants and stromal lesions such as endometrial stromal nodules, and endometrial stromal sarcoma [1]. Our finding of endometrial carcinoma variants to be the most frequent malignant lesion of the endometrium aligns with other studies from Nigeria and other regions of the world [5,10-13,16,24,28-30]. Leiomyoma, a tumor of uterine smooth muscle, and hence not an endometrial neoplasm, can be and is indeed frequently diagnosed in endometrial biopsies, especially if submucous in location [30]. The rate of diagnosis of leiomyoma in our study (3.2%) was higher than the 0.4% reported by Asuzu et al. but lower than the 7.5% reported by Lasmar et al. [5,30]. Ordinarily, it would be expected that our study, based in Nigeria and surveying an exclusively Black population, should have a higher frequency given the reported higher incidence of uterine leiomyoma in Black patient populations [31-33]. The disparity may be attributable to the study design. The referenced studies surveyed leiomyomas diagnosed through other methods, including hysterectomy, ultrasound, and pelvic examination, more suited to diagnosing leiomyomas; while our study analyzed cases in which the samples were obtained via endometrial biopsy only.

Limitations

The major limitation of the study was that it was hospital-based and, therefore, analyzed only the endometrial biopsy samples received at our center. Hence, the findings may not reflect the complete diagnostic spectrum and the true prevalence and incidence of endometrial lesions within the population. Another limitation is the retrospective nature of the study design, which was associated with incomplete data and, hence, possible distortion of the rendered statistics. Inadequate storage facilities and retrieval of archival material, as well as the exclusion of endometrial lesions diagnosed at hysterectomy, also contributed to the non-inclusion of some patient materials that may have influenced the findings of the study.

## Conclusions

Our findings show that the morphological spectrum of endometrial biopsies in Nigerians is generally similar to findings reported from other regions of the world. The lesions were predominantly non-neoplastic and seen in patients in the reproductive age group, while lesions in peri and postmenopausal patients were fewer and more likely to be malignant. The relatively high number of specimens of products of conception may be due to many factors, including inadequate funding and infrastructural, and staffing deficiencies. It may also signify an unacceptably high rate of early pregnancy loss in our setting. Nationwide studies are required to validate this observation. Larger, prospective, multicenter, chromosomal, and cytogenetic studies may then be required to determine genetic predispositions, if any, among Nigerians regarding the incidence of early pregnancy loss. The high proportion of endometrial polyps in non-pregnancy-related biopsies also warrants further studies on possible hormonal influences.
